# Fresh fruit, vegetables, and mushrooms as transmission vehicles for *Echinococcus multilocularis* in Europe: inferences and concerns from sample analysis data from Poland

**DOI:** 10.1007/s00436-016-5015-4

**Published:** 2016-03-18

**Authors:** Lucy J. Robertson, Karin Troell, Ian David Woolsey, Christian M. O. Kapel

**Affiliations:** Parasitology, Department of Food Safety and Infection Biology, Norwegian University of Life Sciences, PO Box 8146 Dep., 0033 Oslo, Norway; Section of Bioinformatics and Molecular Biology, Department of Microbiology, National Veterinary Institute, SE-751 89 Uppsala, Sweden; Section of Organismal Biology, Department of Plant and Environmental Sciences, Faculty of Science, University of Copenhagen, Thorvaldsensvej 40, DK1870 Frederiksberg C, Denmark

**Keywords:** Contamination, *Echinococcus multilocularis*, Foxes, Fruits, Mushrooms, Vegetables

## Abstract

Fresh fruit, vegetables, mushrooms, and other fresh produce are recognised as important vehicles of infection for several foodborne parasites, particularly those with a faecal-oral transmission route and robust environmental transmission stages. Nevertheless, analysis of such foods for parasite transmission stages, even during outbreaks, tends to show only low contamination. *Echinococcus multilocularis* is considered one of the most important foodborne parasites, but there are few studies in which fresh produce or like foods collected in their natural habitat is analysed for contamination with *E. multilocularis* eggs. In this article, we question a recent study from Poland reporting over 23 % of fresh berries, vegetables, and mushroom being highly contaminated with *E. multilocularis* eggs. In particular, it appears unlikely that 20 % of raspberries, which are elevated from ground level, should be exposed to faecal contamination. Additionally, the similar egg contamination of vegetation in forest and plantation environments is surprising considering the preference of the parasite’s most competent intermediate hosts for the latter environment. Furthermore, a lack of specific temporal information is concerning due to the varying infection pressure (and therefore environmental contamination) occurring in definitive hosts over the course of the year. Several important aspects of the study seem to us to have been neglected, and we are concerned that the published data might, if not questioned, lead to incorrect interpretation, and unnecessary losses in the agricultural sector.

Fresh fruit, vegetables, mushrooms, and other fresh produce are recognised as important transmission vehicles for a range of foodborne pathogens (Yeni et al, 2015, Sivapalasingam et al, 2004), including bacteria, viruses, and parasites. A risk-ranking exercise conducted by FAO/WHO in 2012 identified 24 important foodborne parasites globally, of which 11 were considered to have consumption of fresh produce as the transmission route (FAO/WHO, 2014). Among the six parasites that ranked highest, four (*Echinococcus granulosus*, *Echincococcus multilocularis*, *Cryptosporidium* spp., and *Entamoeba histolytica*) had fresh produce as their primary food vehicle and two (*Toxoplasma gondii* and *Taenia solium* (cysticercosis)) as a secondary food vehicle. Although several factors that influence such ranking may differ regionally (e.g. Robertson et al, 2015), fresh produce remains of clear importance as a foodborne parasite transmission vehicle.

Despite the abovementioned ranking of importance, relatively few of the outbreaks where fresh produce has been identified as a vehicle of parasite infection have been documented. For *E. granulosus*, *E. multilocularis*, and *T. solium* (cysticercosis) the lack of identification of outbreaks of infection, or even individual cases of infection, in which fresh produce is definitively identified as the transmission vehicle is assumed to be due to the relatively long incubation period (for *Echinococcus* spp. 10–15 years). For *Toxoplasma*, the clinical picture differs between genotypes, and in Europe, most infections are asymptomatic or sub-clinical. Nevertheless, consumption of fresh escarole (broad-leaved endive) was associated with *Toxoplasma* infection in 11 people, eight of whom developed acute toxoplasmosis, at an industrial plant in Brazil (Ekman et al, 2012).

For *E. histolytica*, the relative fragility of the cysts means that contamination of a food transmission vehicle resulting in infection is probably from a food handler shortly prior to consumption. Thus, extensive or community-wide outbreaks may be less likely to occur. However, again, even individual cases in which fresh produce is definitively identified as the transmission vehicle are lacking.

Among the important foodborne parasites, *Cryptosporidium* spp. stands out as being associated with several well-documented outbreaks in which fresh produce is clearly implicated. These include an outbreak involving 300 cases of cryptosporidiosis from UK in 2009 associated with consumption of pre-cut mixed salad leaves (McKerr et al, 2015) and an outbreak of over 250 cases from Finland in 2012 associated with consumption of frisée salad (Åberg et al, 2015). Notable for both these outbreaks, as well as other outbreaks of cryptosporidiosis associated with fresh produce, is that *Cryptosporidium* oocyst contamination was not detected on the implicated fresh produce. In the UK outbreak, this was because sampling and analysis was not actually conducted due to pragmatic reasons (McKerr et al, 2015). However, in the outbreak from Finland 29, different food samples of various sorts were analysed (Åberg et al, 2015). Various analyses of fresh produce for *Cryptosporidium* contamination have been published over the years, with methods generally based upon or related to the recently published ISO Method (ISO, 2016). These analyses generally indicate a low level of contamination of fresh produce with *Cryptosporidium* oocysts in more industrialised countries (e.g. Dixon et al, 2013; Robertson and Gjerde, 2001), with <10 % contamination, and even when contaminated water is used for irrigation, the number of oocysts per sample is relatively low (Amorós et al., 2010). Higher contamination rates may be found in less industrialised countries, where hygienic infrastructure may be inadequate (e.g. Duedu et al, 2014). It is clear that, despite several relatively extensive outbreaks of foodborne cryptosporidiosis being documented in the literature, particularly from Europe, the contamination of fresh produce for consumption seems to be relatively low level.

Although cases of echinococcosis (*E. multilocularis*, *E. granulosus*) and cysticercosis (*T. solium*) in Europe are relatively few, in other parts of the world they are a serious public health problem. Within Europe, some areas have a considerably higher case burden than others, and studies from Poland have indicated a relatively high number of cases of alveolar echinococcosis even in comparison with neighbouring Baltic countries (Estonia, Latvia, Lithuania) (Torgerson et al 2010). During an assessment of the epidemiological situation in 2012, seven cases were registered, equivalent to 0.018 cases per 100,000 of population (Gołąb and Czarkowski, 2014). In addition, a seroprevalence study conducted in 2011 found three seropositive cases among 172 (1.7 %) inhabitants (aged from 14 to 75 years) from rural Poland (so a selected population that may be more likely to be at risk). Although for none of the seropositive cases were risk factors definitively identified, playing with dogs was suggested as one possibility. In the assessment of the epidemiological situation of echinococcosis and cysticercosis in 2012, risk factors for infection were not identified, but a recommendation was made to intensify educational efforts among the general population without stating how the education would be targeted (Gołąb and Czarkowski, 2014).

Against this background, studies conducted to assess the importance of fresh produce for the transmission of parasites are highly warranted. A recent study was conducted to determine the extent of contamination of fruits, vegetables, and mushroom with *E. multilocularis* in Poland (Lass et al. 2015). As well as providing an indication of the likelihood of food as a transmission route, such a survey might be of use in the educational efforts mentioned above. However, given the low number of cases of human echinococcosis along with our experience from other parasites such as *Cryptosporidium* (for which the number of cases is much higher but contamination of vegetables is relatively moderate), a high degree of contamination would not be expected. One exception, however, might be in specific “hot spots”, where there is a high prevalence of infection among relatively large canid populations. As the authors selected rural areas of Warmia-Masuria Province for their study, which has the highest prevalence (50 %) of infected foxes with *E. multilocularis*, and also with high numbers of worms per intestine (Karamon et al, 2014), this aspect was also considered. Foxes are common in Poland, with the density varying with season, but probably ranging from around 1 per km^2^ to around 0.4 per km^2^ depending on the time of year (lowest at end of winter, highest after birth of kits), and has increased somewhat in recent years (Goszczyński, 1989; Jamrozy, 2008; Mullins et al, 2014). However, the density of foxes remains higher in many urban areas in Europe (Wandeler et al, 2003).

Although *E. multilocularis* eggs are notoriously robust, the transmission stages of other parasites also survive for prolonged periods. Given the relative host population density, along with parasite excretion rates, survival, and available land, it would still seem unlikely that contamination of fresh produce with *E. multilocularis* eggs would be higher than with *Cryptosporidium* oocysts.

Therefore, it was surprising to us to see that the analysis of fresh fruits, vegetables, and mushrooms in the survey from Poland, demonstrated that over 23 % of samples was positive for *E. multilocularis*. Furthermore, the extent of this apparent contamination in each positive sample must also be high as the limit of detection (using PCR) was 100 eggs (Lass et al. 2015). Indeed, the authors suggest that the extent of contamination is probably even higher, due to losses of parasites in the analytical procedure. If these data were correct, then it would suggest that the extent of infection with *E. multilocularis* in the Polish population in this area should be much greater than indicated by the data published. Although the authors state that this is evidence of contamination with *E. multilocularis* eggs and suggest that these data indicate the likelihood of further cases of human infection appearing in the community within the coming decades, they do not accommodate a distinction between viable and non-viable eggs. This may be very relevant when considering infection risks.

We found it particularly surprising that 20 % of samples of raspberries from plantations was found to be contaminated—as, unlike with ground-level plants (mushrooms, salad vegetables) or fruits on low bushes (strawberries, cowberries), it would seem to us unlikely that raspberry fruit, growing relatively high up, would be at much risk of becoming contaminated with faeces from foxes or other canids, or from run-off from contaminated soil. The similar contamination of vegetation from forests and plantations is surprising considering that plantation environments are more likely to favour grassland rodents (*Microtus* spp. and *Arvicola* spp.) that are not common in forest habitats. *Myodes glareolus* and *Apodemus* spp. occur in forest habitats (Giradoux et al, 2003), but evidence suggests these species are significantly inferior hosts for *E. multilocularis* (Woolsey et al, 2015a; Woolsey et al 2015b; Takeiuchi-Strom et al. 2015).

A further concern/limitation is the range of time these vegetation samples were taken. The authors provide vague temporal information as to when these samples were collected (June 2011–September 2012) encompassing all seasons. It has been demonstrated in Zurich (Switzerland) that fox infection rates (and presumably therefore, environmental contamination) vary with season, with a higher percentage of foxes infected during the winter months (Lewis et al, 2014). A breakdown of the proportion of vegetation collected per season would have been valuable especially considering the overlap of growing seasons for certain vegetation (autumn/winter for vegetables, spring/summer for forest fruits). The viability of the eggs would be particularly relevant for any vegetation collected in late summer.

To exclude any contamination of sampled material or laboratory contamination, it would have been advisable to include samples from indoor production that would have addressed unwanted contamination at the laboratory level or comparable samples for a non-endemic area like UK or mainland Norway. Without such negative controls, the work appears less conclusive.

Thus, whilst we applaud the thought behind taking steps to develop methods to analyse fresh produce for contamination with *Echinococcus*, we are concerned that the data produced from this study gives an unrealistic and worrisome indication on the extent of contamination with infective *Echinococcus* eggs. Although clearly fresh produce eaten raw may act as a vehicle for transmission for *Echinococcus*, the inferences to be drawn from the article of Lass et al. (2015) are sufficiently dramatic, that they may either result in people believing fresh produce from this area and eaten raw should be avoided or may result in the arousal of an unfortunate scepticism to the results obtained from other such studies.

## References

[CR1] Åberg R, Sjöman M, Hemminki K, Pirnes A, Räsänen S, Kalanti A, Pohjanvirta T, Caccio SM, Pihlajasaari A, Toikkanen S, Huusko S, Rimhanen-Finne R (2015). *Cryptosporidium parvum* caused a large outbreak linked to frisée salad in Finland, 2012. Zoonoses Public Health.

[CR2] Amorós I, Alonso JL, Cuesta G (2010). *Cryptosporidium* oocysts and *Giardia* cysts on salad products irrigated with contaminated water. J Food Prot. 73(6):1138–1140 10.4315/0362-028x-73.6.1138 20537274

[CR3] Dixon B, Parrington L, Cook A, Pollari F, Farber J (2013). Detection of *Cyclospora*, *Cryptosporidium*, and *Giardia* in ready-to-eat packaged leafy greens in Ontario, Canada. J Food Prot.

[CR4] Duedu KO, Yarnie EA, Tetteh-Quarcoo PB, Attah SK, Donkor ES, Ayeh-Kumi PF (2014). A comparative survey of the prevalence of human parasites found in fresh vegetables sold in supermarkets and open-aired markets in Accra, Ghana. BMC Res Notes.

[CR5] Ekman CC, Chiossi MF, Meireles LR, Andrade Júnior HF, Figueiredo WM, Marciano MA, Luna EJ (2012). Case-control study of an outbreak of acute toxoplasmosis in an industrial plant in the state of São Paulo, Brazil. Rev Inst Med Trop Sao Paulo.

[CR6] Giradoux P, Craig PS, Delattre P, Bao G, Bartholomot B, Harraga S, Qur JP, Raoul F, Wang Y, Shi D, Vuitton DA (2003). Interactions between landscape changes and host communities can regulate *Echinococcus multilocularis* transmission. Parasitol.

[CR7] Gołąb E, Czarkowski MP (2014). Echinococcosis and cysticercosis in Poland in 2012. Przegl Epidemiol.

[CR8] Goszczyński J (1989). Population dynamics of the red fox in central Poland. Acta Theriol (Warsz).

[CR9] ISO (2016). ISO 18744; Microbiology of the food chain -- Detection and enumeration of *Cryptosporidium* and *Giardia* in fresh leafy green vegetables and berry fruits).

[CR10] Jamrozy G (2008). Carnivores, even-toed ungulates, lagomorphs and large rodents in Polish national parks. Ann Zool Fenn.

[CR11] Karamon J, Kochanowski M, Sroka J, Cencek T, Różycki M, Chmurzyńska E, Bilska-Zając E (2014). The prevalence of *Echinococcus multilocularis* in red foxes in Poland—current results (2009-2013). Parasitol Res.

[CR12] Lass A, Szostakowska B, Myjak P, Korzeniewski K (2015). The first detection of *Echinococcus multilocularis* DNA in environmental fruit, vegetable, and mushroom samples using nested PCR. Parasitol Res.

[CR13] Lewis FI, Otero-Abad B, Hegglin D, Deplazes P, Torgerson PR (2014). Dynamics of the force of infection: insights from *Echinococcus multilocularis* infection in foxes. PLoS Negl Trop Dis.

[CR14] McKerr C, Adak GK, Nichols G, Gorton R, Chalmers RM, Kafatos G, Cosford P, Charlett A, Reacher M, Pollock KG, Alexander CL, Morton S (2015). An outbreak of *Cryptosporidium parvum* across England & Scotland associated with consumption of fresh pre-cut salad leaves, May 2012. PLoS One.

[CR15] Mullins J, McDevitt AD, Kowalczyk R, Ruczyńska I, Górny M, Wójcik JM (2014). The influence of habitat structure on genetic differentiation in red fox populations in north-eastern Poland. Acta Theriol (Warsz).

[CR16] Robertson LJ, Gjerde B (2001). Occurrence of parasites on fruits and vegetables in Norway. J Food Prot.

[CR17] Robertson LJ, Goyal K, Sehgal V (2015). An Indian multicriteria-based risk ranking of foodborne parasites. Food Res Int.

[CR18] Sivapalasingam S, Friedman CR, Cohen L, Tauxe RV (2004). Fresh produce: a growing cause of outbreaks of foodborne illness in the United States, 1973 through 1997. J Food Prot.

[CR19] Takeiuchi-Strom N, Woolsey ID, Jensen PM, Fredensborg BL, Pipper CB, Kapel CMO (2015). Predictors of *Echinococcus multilocularis* prevalence in definitive and intermediate hosts: a meta-analysis approach. J Parasitol.

[CR20] Torgerson PR, Keller K, Magnotta M, Ragland N (2010). The global burden of alveolar echinococcosis. PLoS Negl Trop Dis.

[CR21] Wandeler P, Funk SM, Largiadèr CR, Gloor S, Breitenmoser U (2003). The city-fox phenomenon: genetic consequences of a recent colonization of urban habitat. Mol Ecol.

[CR22] Woolsey ID, Bune NET, Jensen PM, Deplazes P, Kapel CMO (2015). *Echinococcus multilocularis* infection in the field vole (*Microtus agrestis*): an ecological model for studies on transmission dynamics. Parasitol Res.

[CR23] Woolsey ID, Jensen PM, Deplazes P, Kapel CMO (2015). Establishment and development of *Echinococcus multilocularis* metacestodes in the common vole (*Microtus arvalis*) after oral inoculation with parasite eggs. Parasitol Int.

[CR24] Yeni F, Yavaş S, Alpas H, Soyer Y (2015). Most common foodborne pathogens and mycotoxins on fresh produce: a review of recent outbreaks. Crit Rev Food Sci Nutr.

